# Mild form of Zellweger Spectrum Disorders (ZSD) due to variants in *PEX1*: Detailed clinical investigation in a 9-years-old female

**DOI:** 10.1016/j.ymgmr.2020.100615

**Published:** 2020-06-20

**Authors:** Maria Rosaria Barillari, Marianthi Karali, Valentina Di Iorio, Maria Contaldo, Vincenzo Piccolo, Maria Esposito, Giuseppe Costa, Giuseppe Argenziano, Rosario Serpico, Marco Carotenuto, Gerarda Cappuccio, Sandro Banfi, Paolo Melillo, Francesca Simonelli

**Affiliations:** a Division of Phoniatrics and Audiology, Department of Mental and Physical Health and Preventive Medicine, University of Campania "Luigi Vanvitelli", Via L. De Crecchio 4, 80138 Naples, Italy; b Telethon Institute of Genetics and Medicine, Pozzuoli, Via Campi Flegrei 34, 80078 Pozzuoli, Italy; c Department of Precision Medicine, University of Campania "Luigi Vanvitelli", Via L. De Crecchio 7, 80138 Naples, Italy; d Eye Clinic, Multidisciplinary Department of Medical, Surgical and Dental Sciences, University of Campania Luigi Vanvitelli, Via Pansini 5, 80131 Naples, Italy; e Dental Clinic, Multidisciplinary Department of Medical, Surgical and Dental Sciences, University of Campania “Luigi Vanvitelli”, Via L. De Crecchio 6, 80138 Naples, Italy; f Pediatric Dermatology, Dermatology Unit, University of Campania Luigi Vanvitelli, Via Pansini 5, 80131 Naples, Italy; g Clinic of Child and Adolescent Neuropsychiatry, Department of Mental Health, Physical and Preventive Medicine, University of Campania "Luigi Vanvitelli", Via Pansini 5, 80131 Naples, Italy; h Department of Translational Medicine, Section of Paediatrics, Federico II University, Via Pansini 5, 80131 Naples, Italy

**Keywords:** Sensorineural hearing loss, Retinitis pigmentosa, Enamel defects, *PEX* genes, Peroxisomal biogenesis disorders, Mild Zellweger syndrome, PBD, Peroxisomal biogenesis disorders, SNHL, sensorineural hearing loss, RP, retinitis pigmentosa, PEX, peroxin, ZSD, Zellweger spectrum disorder, ZS, Zellweger Syndrome, HS, Heimler syndrome, BCVA, Best Corrected Visual Acuity, GVF, Goldmann Visual Field, ERG, full-field electroretinogram, OCT, optical coherence tomography, FAF, color fundus and fundus autofluorescence, TEOAE, Transient-Evoked Otoacustic Emission, ABR, Auditory Brainstem Responses, PTA, Pure Tone Average, WISC-IV, Wechsler Intelligence Scale for Children (4th Edition), CDI, Children’s Depression Inventory, VLCFA, Very Long Chain Fatty Acid

## Abstract

Peroxisomal biogenesis disorders (PBD) are rare autosomal recessive disorders with various degrees of severity caused by hypomorphic mutations in 13 different peroxin (PEX) genes. In this study, we report the clinical and molecular characterization of a 9-years-old female presenting an apparently isolated pre-lingual sensorineural hearing loss (SNHL) and early onset Retinitis Pigmentosa (RP) that may clinically overlap with Usher syndrome. Genetic testing by clinical exome sequencing identified two variants in *PEX1*: the missense variant c.274G > C; p.(Val92Leu) that was already reported in a PBD patient, and the variant c.2140_2145dup; p.(Ser714_Gln715dup) which is a novel, non-frameshift variant, absent in control databases. On the basis of the molecular analysis, a thorough clinical examination revealed nail and dental abnormalities, a mild cognitive impairment, learning disabilities and poor feeding, apart from the retinal and audiological features initially identified. The clinical and molecular findings led us to the diagnosis of a mild form of PBD. This study further emphasizes that mild forms of PBD can be a differential diagnosis of Usher syndrome and suggests that patients with mild cognitive impairment associated to visual and hearing loss should perform a comprehensive mutation screening that includes *PEX* genes.

## 1 Introduction

Peroxisomal biogenesis disorders (PBDs) are a group of autosomal recessive disorders caused by mutations in one of the thirteen Peroxin (*PEX*) genes. PBDs are caused by partial or generalized defects in peroxisome biogenesis [1]. These organelles are present in almost all eukaryotic cells and play an indispensable role in many biochemical pathways including the metabolism of branched chain and very long chain fatty acids, ether lipids, polyamines, amino acids and glyoxylate [2,3]. Peroxisomal dysfunction leads to multisystem disease that includes neurological, visual and hearing symptoms [4].

PBDs are divided into two main types: the Rhizomelic Chondrodysplasia Punctata type 1 and the Zellweger spectrum disorder (PBD-ZSD) which ranges from severe (Zellweger Syndrome, ZS) to intermediate (Neonatal Adreno-LeukoDystrophy) and mild (Infantile Refsum Disease) phenotypes [5]. The PBD-ZSD is a clinically heterogeneous group with a continuum of disease severity. In particular, the clinical presentation of the most severe form, namely ZS (OMIM: 614872), is characterized by craniofacial abnormalities, neuronal migration defects, leukodystrophy, absence of language, cognitive and psychomotor delay, renal and liver diseases, hypotonia, hearing loss and vision problems including cataracts and/or retinal abnormalities [6]. Children with this condition do not develop properly and usually die before one year of age [7], while patients with intermediate and mild phenotypes can live into adulthood because their clinical features (e.g. sensorineural hearing loss, retinal diseases, leukodystrophy and cognitive delay) are less pronounced compared to those of ZS. Pathogenic variants in *PEX1* are the most common cause of PBD-ZSD and have been associated with various degrees of disease severity [[8], [9], [10], [11]]. On the other hand, the Heimler Syndrome (HS) is considered the mildest end of PBD-ZSD spectrum of disorders [12]. HS is caused by hypomorphic mutations in *PEX1* and *PEX6* genes and is characterized by severe to profound bilateral sensorineural deafness, enamel defects and nail abnormalities such as Beau's lines and punctate leukonychia [[12], [13], [14], [15]]. Macular dystrophy has also been reported in the context of the HS^16^.

The clinical heterogeneity of PBD-ZSD renders patients' diagnosis challenging, especially in the case of very mild, late-onset forms that may overlap with other syndromic phenotypes, in particular Usher syndrome. This may delay diagnosis and proper management of the disease.

Here, we report the clinical and molecular characterization of a 9-years-old female presenting an apparently isolated sensorineural hearing loss (SNHL) and early onset atypical Retinitis Pigmentosa (RP). Clinical exome sequencing identified two biallelic variants in the *PEX1* gene. A thorough clinical reevaluation led to the diagnosis of a mild form of PBD-ZSD. We therefore suggest that patients with mild cognitive impairment associated to visual and hearing loss should perform a comprehensive mutation screening that includes *PEX* genes.

## 2 Materials and methods

Written informed consent for research and publication was obtained by the family prior to participation of the subject to the current study. All the procedures of this study were in complete accordance with the Declaration of Helsinki on ethical principles for medical research involving human subjects (2014).

The patient underwent a multidisciplinary clinical examination. It included ophthalmological and audiological tests as well as pediatric, dental, dermatological and neuropsychological evaluation and standard biochemical tests.

### 2.1 Ophthalmological evaluation

Best Corrected Visual Acuity (BCVA), slit lamp anterior segment examination, Goldmann Visual Field (GVF), fundus examination, full-field electroretinogram (ERG), optical coherence tomography (OCT), color fundus and fundus autofluorescence (FAF) were performed.

### 2.2 Audiological evaluation

The audiological assessment was obtained by the use of Otoacustic Emission (OAE), in terms of Transient-Evoked Otoacustic Emission (TEOAE), Auditory Brainstem Responses (ABR), liminar pure tone audiometry with evaluation of the Pure Tone Average (PTA) and Impedance test composed by tympanometry and stapedial reflexes.

### 2.3 Neuropsychological evaluation

This clinical examination was performed by a trained child psychiatrist and included an interview with the patient, clinical observation of her behavior and the administration of validated tests and questionnaires such as SCARED and WISC-IV.

### 2.4 Dental evaluation

An extra- and intra-oral examination was performed to evaluate the oral mucosal integrity and teeth status. An X-ray orthopantomography was combined with the clinical evaluation to identify any dental agenesis, dental malocclusions and other abnormalities in the shape and/or structure of the teeth.

### 2.5 Dermatological examination

A total body examination was performed including inspection of nails and hair. Further details about the above-mentioned tests are provided in Supplementary Materials.

### 2.6 Biochemical analysis

Measurement of plasmatic Very Long Chain Fatty Acid (VLCFA) and branched-chain fatty acid was performed by a specialized laboratory using capillary gas chromatography/mass spectrometry of pentafluorobenzyl bromide fatty acid esters as described elsewhere [17,18].

### 2.7 Clinical exome sequencing and segregation analysis

The proband's genomic DNA sample underwent a panel-based Next Generation Sequencing using the ClearSeq Inherited Disease Panel (Agilent) that allows the screening of more than 2700 genes known to cause human inherited disorders. Sequencing data were analysed as previously described [19]. Selected variations were validated by Sanger sequencing in the patient and parents to assess proper segregation. Further details are provided in Supplementary Materials.

## 3 Results

The patient was born at 40 weeks of gestation to unrelated parents with no prior pregnancies. The Apgar index at birth was 8/10. No dysmorphic features were evident, and no low muscle tone was reported.

At 3 months of age she presented a bilateral sensorineural hearing loss, diagnosed by the use of TEOAE and ABR. The auditory deficit was treated by digital hearing aids and speech therapy. Annual audiological follow-ups were regularly performed.

The patient was clinically evaluated by our multidisciplinary team for the first time at the age of 9 because of a suspicion of an inherited retinal disease associated to SNHL.

### 3.1 General pediatric evaluation

In the last pediatric evaluation performed at 9 years of age the subject had a height of 128.6 cm (−2.17 SD), an OFC of 49.3 cm (−3.7 SD) and weighed 22.9 Kg (−2.6 SD). Her low weight was associated also with a significant food selectivity and poor feeding for which a specific behavioral feeding program was indicated.

The values of the routine hematochemical analysis were within the normal range. Liver function tests showed normal levels of glutamic oxaloacetic transaminase (GOT) and serum glutamic-pyruvic transaminase (GPT) at 9 years, respectively of 21.58 U/L (N·V: < 42 U/L) and 21.23 U/L (N·V: < 41). Similarly, alkaline phosphatase, albumin, creatinine and total protein values were normal. Total bile acids were normal at the age of 9 while total bilirubin resulted weakly elevated at the last check (1.49 mg%; N·V: <1).

Abdominal ultrasounds revealed no major renal or liver abnormalities.

### 3.2 Ophthalmological findings

**Fig. 1:**
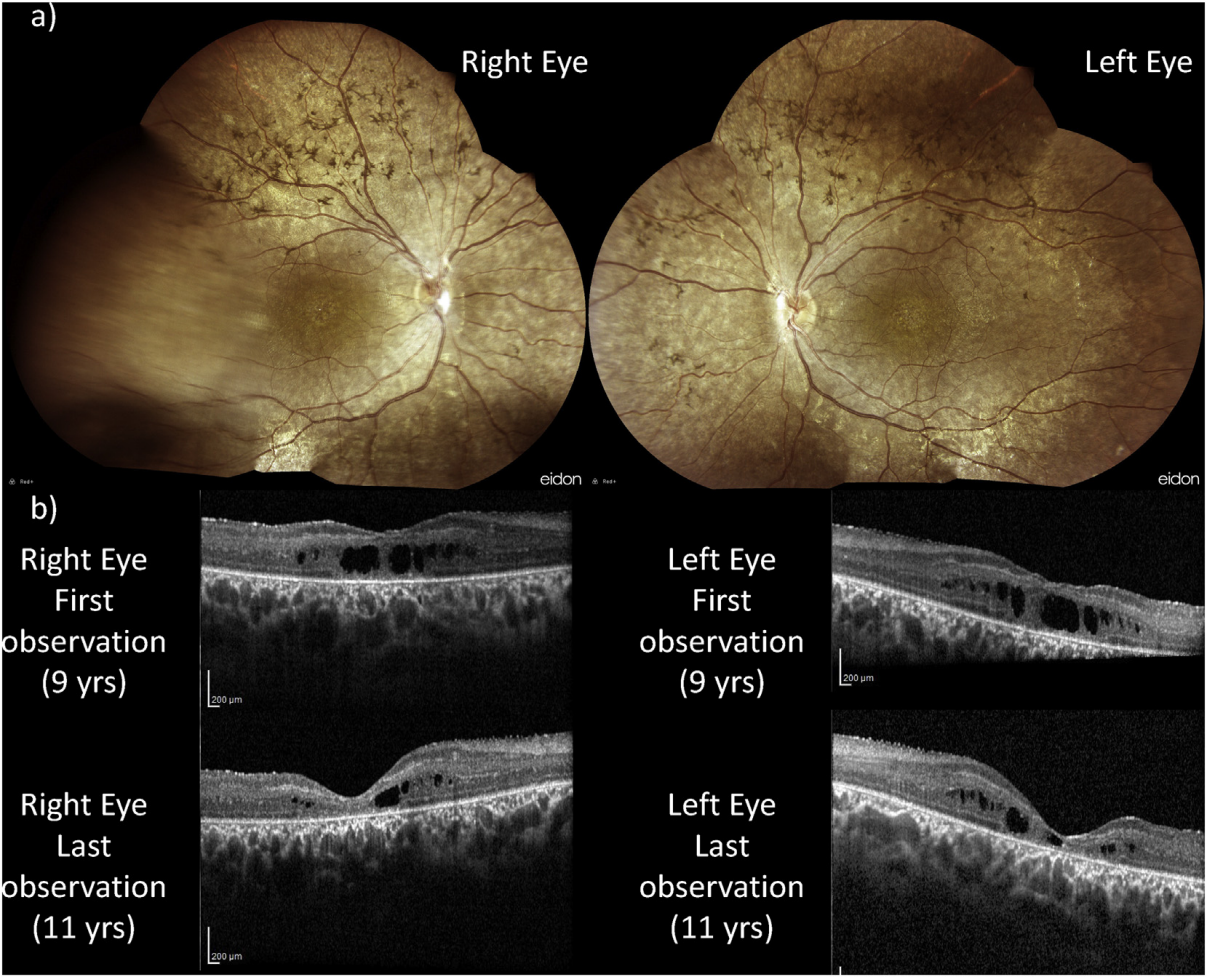
Fundus imaging (a) and OCT scans (b) of the reported case.

At the anamnestic evaluation, the subject referred night-blindness in the last year. BCVA was 20/120 in both eyes. Fundus examination revealed a normal optic disk, punctiform lens opacities, dystrophy of Retinal Pigmented Epithelium (RPE) with bone spicule-shaped pigment deposits arranged within and beyond the vascular arcades with a normal appearance of the far periphery and macular dystrophy (Fig. 1a). GVF showed a constricted visual field. Specifically, the average radius was 17° and 27° using the III4e target stimulus size and 30° and 40° using the V4e target stimulus size in the right and left eye, respectively. OCT scans showed RPE dystrophy with loss of the EZ band in both eyes and revealed multiple inner retinal cystoid spaces (with a mean macular thickness of 260 μm and 293 μm in the right and left eye, respectively) (Fig. 1b). FAF imaging revealed hyper- and hypo-autofluorescent dots in the posterior pole and beyond the temporal vascular arcades sparing the fovea in both eyes. Dark-adapted 0.01 ERG responses were below noise level, whereas dark-adapted 3.0 responses were subnormal in both eyes with a b/a ratio < 1. Light-adapted ERG responses were subnormal in both eyes. Given the presence of cystoid spaces, the patient was prescribed treatment with oral acetazolamide (250 mg/die) and was regularly followed up. Over the two-year follow-up period, we recorded a reduction of cystic spaces (with mean macular thickness of 185 μm and 208 μm in the right and left eye, respectively) without significant changes in BCVA and in GVF (Fig. 1b). Taken together, the ophthalmological findings confirmed an ocular phenotype compatible with RP complicated by macular cystoid edema.

OCT scans over a two-year follow-up showed an improvement of the macular edema in the patient following treatment with acetazolamide.

### 3.3 Audiological findings

There was no history of sensorineural deafness on either side of the family. The patient has one younger sister with a normal audiometric threshold. Parents reported that the child underwent the universal newborn hearing screening program (performed by law in Italy) twenty-four hours after birth with normal results (a document reporting the results of TEOAE was not available). At 3 months of age, the general practitioner suspected hearing loss and recommended a specific audiological evaluation which was performed by ABR. This test, considered as the gold standard for the early diagnosis of childhood deafness, showed an abnormal morphology and latency of the auditory brain response waves. The latency of the I, III and V waves was increased and only the V wave was observed up to 60 dB hearing level bilaterally. This finding suggested an auditory deficit characterized by severe SNHL in both ears.

**Fig. 2:**
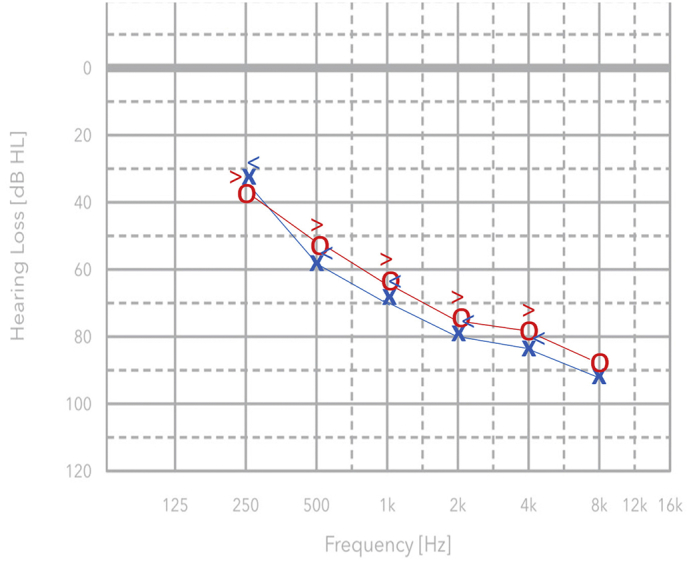
Pure tone audiometry (air and bone conduction) in the right ear (in red) and in the left ear (in blue) in the frequency range between 250 Hz - 8 KHz. Down-sloping audiogram with severe bilateral sensorineural hearing loss is more evident in the higher frequencies.

The impedance test showed a Type “A” tympanogram bilaterally, considered normal, and the presence of the stapedial reflexes (SR) in ipsi and contra in the entire frequency range. The Metz recruitment test, i.e. the gap between the acoustic reflex threshold and the pure-tone audiometry hearing threshold level, was positive and indicated a cochlear SNHL. Subsequent checks performed at the age of 6 and 9 months by ABR confirmed the SNHL diagnosis prompting for an early rehabilitation treatment by digital hearing aids and speech therapy that started at 10 months of age. The patient has been regularly undergoing an annual audiological check from 2008 to date. The most recent ones, performed at the age of 9 and 10, confirmed the presence of bilateral and symmetric SNHL of a severe degree (according to World Health Organization - Grades of Hearing Impairment) that does not appear to be progressive over time, with a “sloping audiogram” and a PTA (without hearing device) around 70 dB HL (Fig. 2). Further annual follow-ups are needed to monitor the hearing impairment over time.

### 3.4 Phoniatric and neuropsychological findings

A phoniatric and neuropsychological assessment was performed as learning disabilities were referred by the patient's teacher and parents. The administration of ad hoc *tests* revealed a deficit of language skills both in input and output. The obtained results were below the average expected for this age (< 2 SD) and indicated a global immaturity of speech abilities as well as inadequate capacities of phonological discrimination, lexical and grammar understanding.

Phonological and metaphonological skills (particularly the analytical ones) as well as the verbal comprehension were severely compromised compared to the other children in the same age-group This suggests that the child had learning disabilities, especially with regards to reading and writing. Working memory abilities and short-term verbal memory abilities were compromised compared to average degrees. For this reason, a further clinical examination was recommended in order to evaluate the presence of an intellectual disability.

Neuropsychological evaluation, according to the results of behavioral observation and clinical interviews, showed that the patient was affected by Separation anxiety (revealed by SCARED score) that impacts on school performance. The scores obtained by the administration of the WISC-IV (IQ total score = 65) underline the presence of a mild intellectual disability. In light of these findings, the learning disabilities observed in the patient (i.e. in writing and reading) can be considered secondary to the cognitive delay.

Magnetic resonance imaging (MRI) of the brain did not reveal any signs of leukodystrophy or other significant brain abnormalities.

### 3.5 Molecular analysis identifying biallelic variants in PEX1

**Fig. 3:**
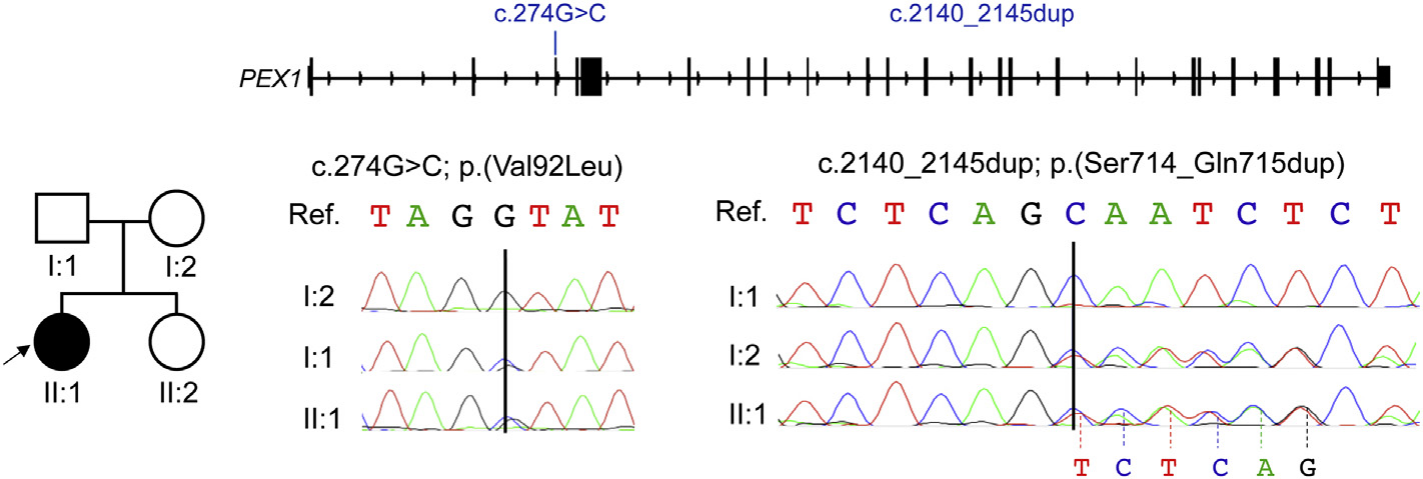
Biallelic variants identified in *PEX1*.

The genetic analysis revealed the presence of two variants of uncertain significance (VUS) in *PEX1* (NM_000466). The identified variants were present in heterozygosity in the proband and their frequency in population databases (e.g. gnomAD, the ExAC database, the 1000 Genomes project) was compatible with a possible pathogenic role. The missense variant c.274G > C; p.(Val92Leu) is predicted to interfere with a canonical splice site (cadd-13 score = 24.6) and was reported in a homozygous state in a patient with atypical Zellweger syndrome [20]. The second variant c.2140_2145dup; p.(Ser714_Gln715dup) is a novel non-frameshift duplication of six nucleotides predicted to introduce a third copy of a Ser-Gln repeat at amino acid position 715 (UniProtKB: O43933). Both variants were validated by Sanger sequencing in the proband and segregated properly in the unaffected parents, confirming their presence *in trans* in the patient (i.e. p.(Val92Leu) on the paternal allele and p.(Ser714_Gln715dup) on the maternal one), consistent with the recessive pattern of inheritance of *PEX1* variants (Fig. 3).

*PEX1* is responsible for ZSD-PBD and HS. Therefore, the identification of these variants in *PEX1* prompted us to reexamine the patient's phenotype in search for clinical features that may have been missed at first evaluation.

Sanger sequencing traces indicate compound heterozygosity of the two variants in exon 3 and exon 13 in the patient (II:1) and heterozygosity in the unaffected parents (I:1, I:2). In exon 13, the duplicated bases of the c.2140_2145dup variant are connected with a dashed line to the corresponding peaks in the proband's chromatogram. Arrow indicates the proband. Ref; Reference sequence.

### 3.6 Laboratory analysis and VLCFA assay

Routine serum VLCFA and branched-chain fatty acid (phytanic and pristanic acid) measurements were performed to check for defects in the metabolism of fatty acids, given the role of peroxins in this process. The proband was not on special diet and fasting serum samples were collected. The majority of the values were within the normal range, with C26:0 slight increase (Table 1) [21].

**Table 1:** Main results of serum standard biochemical markers in the patient.

Serum VLCFA	Patient	Normal control range
C22:0	57.9 μmol/L	26.5–75.3 μmol/L
C24:0	44.9 μmol/L	24.9–73.0 μmol/L
C26:0	1.130 μmol/L^a^	0.460–0.960 μmol/L
C24:0/C22:0	0.77	0.62–1.01
C26:0/C22:0	0.019	0.008–0.026
Pristanic acid	0.13 μmol/L	Traces - 1.50 μmol/L
Phytanic acid	1.76 μmol/L	Traces - 7.0 μmol/L
Pristanic/Phytanic Ratio	0.07	0.01–0.60 μmol/L

a Out-of-normal-range value.

VLCFA plasma concentration may vary, with subjects demonstrating normal or only modest increases, especially in milder or atypical forms of PBDs [22,23]; moreover normal or slightly elevated results of routine serum VLFCA and phytanic acid in patients affected by mild PBDs have already been described by other authors [20,24,25].

Anyway we have to consider that the value of C26:0 obtained in our patient can be due to nonspecific factors and, for this, further immunocytochemical studies in cultured fibroblasts (considered a more sensitive indicator for a mild PBD) would be performed in the future as well as measuring plasma pipecolic acid and the bile acid intermediates dihydroxycholestanoic (DHCA) and trihydroxycholestanoic (THCA) may be helpful.

### 3.7 Dental findings

**Fig. 4:**
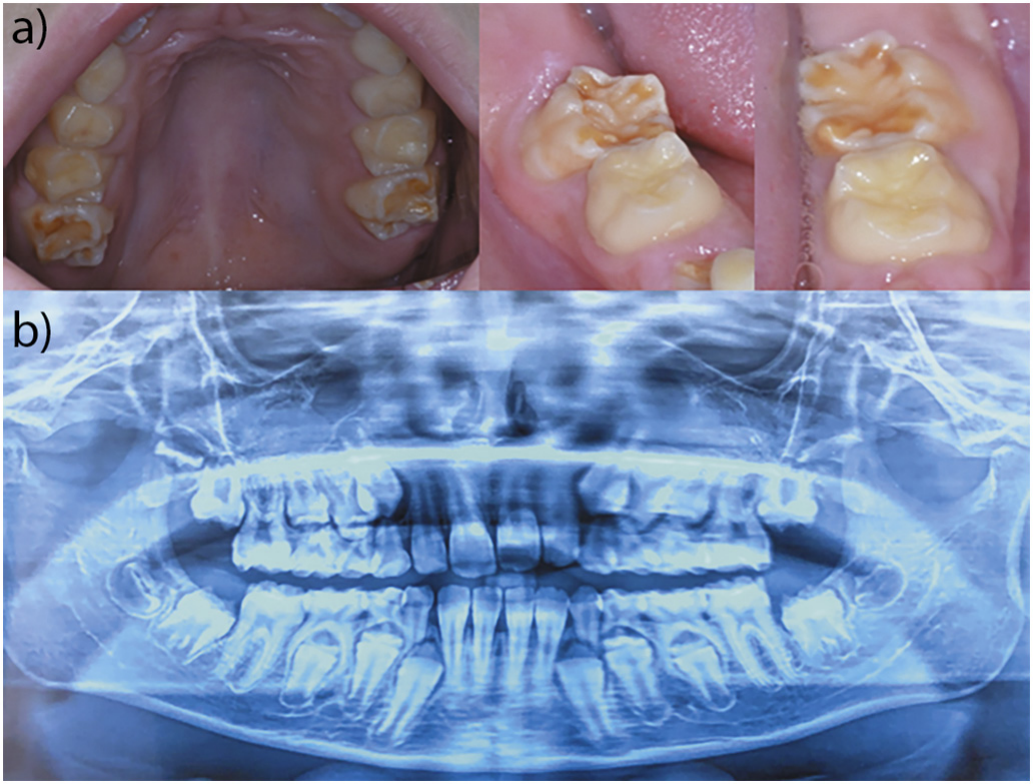
Main dental findings of the reported case. a) High-arched palate and visible enamel hypoplasia of the first permanent upper molars; further chromatic and structural enamel alterations of the first permanent lower molars b) Orthopantomography reveal the presence of all the permanent teeth expected, lack of taurodontism, nor agenesis. The cusps of the permanent canines and premolars, particularly of the mandible, seem thinner and, presumably hypoplastic.

At the extraoral examination, on the frontal plane, the patient showed a symmetrical face both on transverse and vertical planes, with competent lips. At sagittal examination, the profile was straight. The intraoral examination revealed a mixed dentition, as expected at this age [26]. In particular, the upper and lower permanent incisors and the first permanent molar were present. The remaining dentition consisted of primary teeth (canines and molars), except for the two first primary inferior molars, which were exfoliated (as reported by the patient) and replaced by barely visible, thin, erupting cusps. Several enamel and structural defects of various degree affected the permanent teeth [27] (Fig. 4a).

Specifically, the upper central incisors displayed white spots on the vestibular surfaces. Moreover, the occlusal areas of the first permanent molars and the mesial-vestibular margin of the inferior right second incisor were affected by enamel hypoplasia. These appeared yellowish and had an irregular shape at the cusps. The erupting first lower premolars also seemed to have similar defects.

The X-ray orthopantomography (Fig. 4b) revealed no agenesis, since all the permanent teeth were present at a variable degree of maturation and root formation, including the four buds of the third molars. The anatomy of the roots of the permanent teeth did not show any obvious abnormalities. However, the enamel density of canines and premolars appeared reduced and their cusps were slightly hypoplastic. The pulp chambers were dimensionally enlarged as expected at that age, yet not excessively big as in taurodontic teeth, as previously reported [28,29]. On the vertical plane, the occlusion was altered by the presence of an anterior open bite due to mouth breathing reported by the patient. The same behavior was responsible for the discrepancies observed on the transversal plane, between maxilla and mandible, namely a reduced bilateral overjet and a high-arched palate. Oral mucosae were healthy and normochromic. Tongue and teeth were covered by a visible layer of dental plaque, which was subsequently removed by professional oral hygienist.

Overall, the structural and chromatic dental defects pointed towards the diagnosis of enamel defects with hypomineralization, particularly affecting incisors and molars.

### 3.8 Dermatological findings

**Fig. 5:**
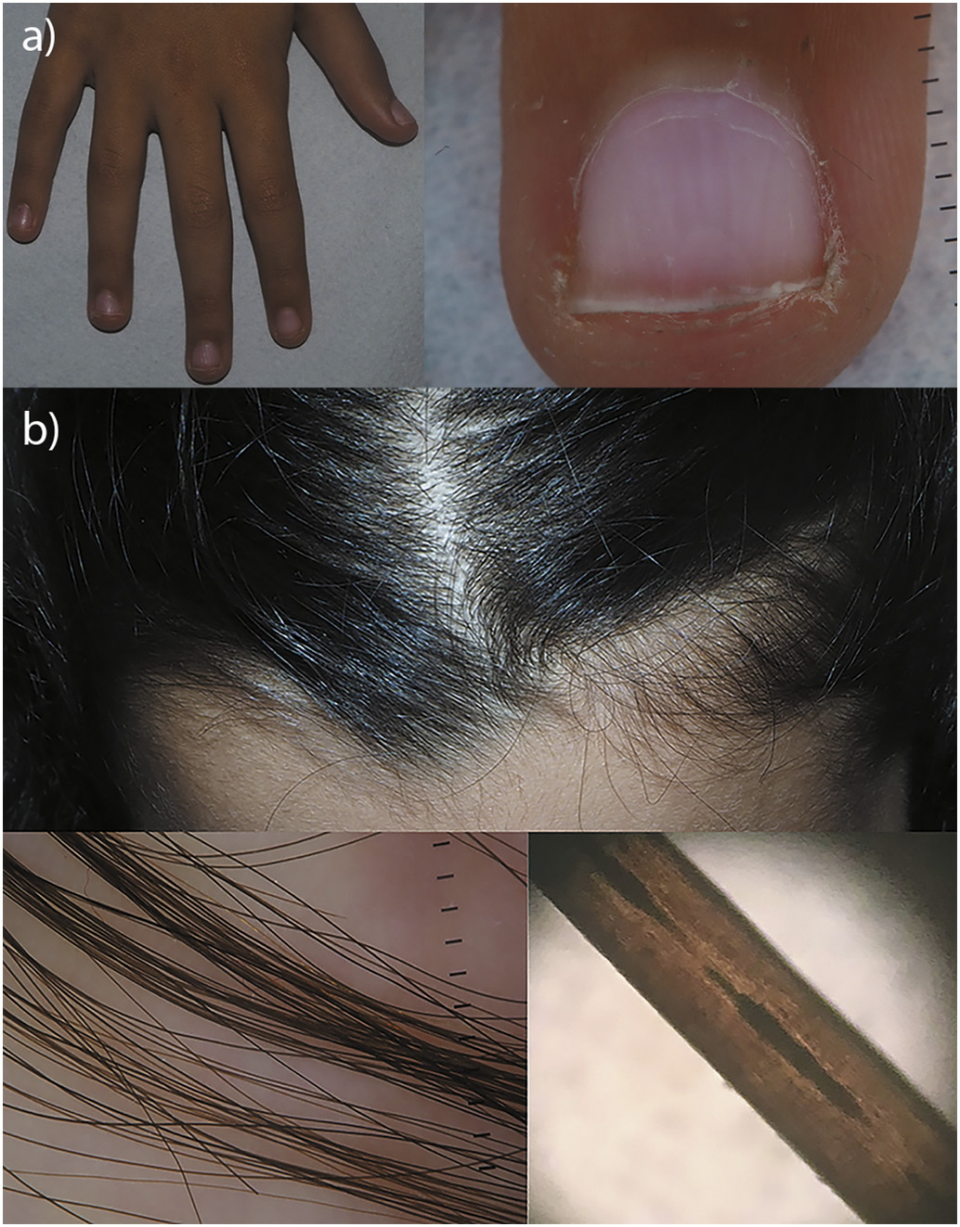
Dermatological findings of the reported case. a) Leukonychia partialis of all fingernails sparing the distal margin of the nail plates, better magnified by onychoscopy in the right caption b) Clinically detectable banding of hair, confirmed by trichoscopy and light microscopy, showing abnormal cavities in the cortex of the hair shaft typical of pili annulati.

Total body skin examination revealed a true leukonychia partialis equally affecting all fingernails and minimally sparing the distal edges of each nail. Leukonychia was confirmed by onychoscopy showing a whitish discoloration of nail plates which did not disappear after pressure on the nail matrix (Fig. 5a). Moreover, a careful examination of scalp hair showed an alternation of light and dark bands of hair shafts. This was confirmed by trichoscopy and corresponded to abnormal cavities in the cortex of the hair shaft compatible with the diagnosis of pili annulati (Fig. 5b).

## 4 Discussion

In this report, we employed a multidisciplinary approach to characterize in detail the clinical phenotype of a pediatric patient with a mild form of PBD-ZSD.

The patient was referred to our clinic with an apparently isolated pre-verbal SNHL and night blindness. The full ophthalmological evaluation revealed a pericentral form of RP complicated by bilateral macular edema. The ocular and audiological phenotype along with the absence of dysmorphic features pointed towards a preliminary clinical diagnosis of Usher syndrome. However, the patient's phenotype of pre-puberal RP and early onset, severe SNHL did not fit well in the spectrum of Usher disease. This was because in Usher type 1 the presence of pre-puberal RP is associated with congenital SNHL of a profound degree, whereas in Usher type 2 a moderate-to-severe (generally congenital) SNHL is normally associated with post-puberal RP.

In parallel, genetic testing by clinical exome sequencing did not reveal variants in genes that cause Usher syndrome, but identified biallelic variants in *PEX1*, a gene responsible for peroxisome biogenesis disorders. The two identified variants were classified as VUS according to the American College of Medical Genetics and Genomics (ACMG) guidelines. The p.(Ser714_Gln715dup) variant is novel and absent from reference databases. Its translation introduces a third copy of a Ser-Gln pair in a small repeat region of PEX1. The second mutation is a missense variant (p.(Val92Leu)) predicted in silico to interfere with a canonical acceptor splice site. We believe that both variants are likely to be pathogenic since they were found *in trans* (as confirmed by segregation analysis) in a patient presenting a clinical phenotype compatible with a peroxisome biogenesis disorder. Future in vitro studies should experimentally verify the functional consequences of the identified variants. In support of its potential causative role, the variant p.(Val92Leu) has been previously described in homozygous state in a Turkish patient with a non-classical ZS, although it was not specified whether zygosity was confirmed by segregation in the parents [20]. This patient, contrary to the case reported herein, presented several dysmorphic features (e.g., large fontanelle, wide sutures, high forehead, broad nasal bridge, external ear deformity and sickle foot), hypotonia, severe psychomotor retardation but no ocular findings [20]. A possible explanation for the absence of ocular findings could be attributed to the patient's age at examination (i.e. 2 years old), also considering that our case reported the first symptoms of visual impairment at the age of 8 years.

The similarity between Usher syndrome and mild phenotypes of PBD-ZSD has been previously reported [21,[30], [31], [32]]. We, therefore, pursued a comprehensive clinical assessment to differentiate between the two possibilities. Neuropsychological assessment of the patient revealed the presence of a mild cognitive impairment, a common feature in the spectrum of peroxisome disorders. Brain MRI did not reveal generalized defects. Moreover, the patient had a lower weight compared with normal population associated with a significant food selectivity and poor feeding, features that have been described in intermediate-mild forms of ZSD [1,5]. Oral examination showed minor oral abnormalities, in terms of enamel defects, as previously described in cases of intermediate-milder PBD-ZSD [5]. The dermatological findings present in the patient (i.e. leukonychia, Beau's lines and hair defects,) are not considered as diagnostic criteria in the spectrum of PBD-ZSD. Nevertheless, mutations in *PEX1* and *PEX6* genes may determine nail and hair defects [31]. Taking into account both the clinical and genetic findings, we formulated a diagnosis of a mild PBD-ZSD. Plasmatic VLCFA levels were overall normal, except for a slight increase of C26:0. This was in line with previous studies showing that individuals with very mild/mild PBDs do not necessarily demonstrate significantly altered values in VLCFA metabolic screening tests as observed for patients with severe forms of ZSD [[33], [34], [35]]. Unfortunately, measurement of C26:0-lysoPC, which was recently proposed as a novel, sensitive serum biomarker for the diagnosis of mild PBD-ZSD [1], could not be performed in the patient, as well as skin biopsy and immunocytochemical studies in cultured fibroblasts as mentioned above.

## 5 Conclusions

In conclusion, we report here a case of mild form of PBD-ZSD characterized by slight abnormalities of VLCFA, early onset SNHL, atypical RP, enamel defects, nail abnormalities, minor feeding problems and mild cognitive impairment in absence of hypotonia, dysmorphic features and other major abnormalities. The association of hearing loss, enamel defect and nail abnormalities, with or without macular dystrophy, has been described also in Heimler syndrome (HS), which is considered the mildest form known to date in the spectrum of PBD [[12], [13], [14], [15]]. Although these clinical features are observed in our patient, the presence of a mild cognitive impairment and feeding problems renders her phenotype more compatible with a mild form of ZSD [1] which has a slightly more severe clinical presentation compared to the typical Heimler syndrome [[13], [14], [15], [16],^22^,^31^].

Based on the presented case, we recommend that patients with visual and hearing impairment perform a comprehensive mutation screening that includes the *PEX* genes*.* The molecular analysis can be instrumental for the early identification of patients with mild forms of PBD-ZSD that may overlap with other syndromic diseases. Moreover, the timely and proper diagnosis of these rare cases requires the combined effort of a multidisciplinary team of clinicians. Following initial diagnosis, periodic multidisciplinary follow-ups are required to monitor disease progression and ensure appropriate disease management.

## Declaration of Competing Interest

None of the authors has a conflict of interest.

## Appendix A Supplementary data

Supplementary material Image 1
