# Supplementary material for: Mild form of Zellweger Spectrum Disorders (ZSD) due to variants in PEX1: Detailed clinical investigation in a 9-years-old female
Source: Mol Genet Metab Rep. 2020 Jun 20;24:100615. doi: 10.1016/j.ymgmr.2020.100615 (PMC7306489; doi:10.1016/j.ymgmr.2020.100615)
Supplement: Supplementary file 1 — Supplementary material [file mmc1.docx]

*Ophthalmological evaluation*

BCVA was measured using the Snellen chart. GVF was measured by moving the III4e and V4e stimulus target on a calibrated standard Goldmann perimeter by an experienced ophthalmic technician and was analysed as previously published^1^. Full-field ERG was recorded by corneal contact lens electrodes with a Ganzfeld stimulator (Roland Consult, Brandenburg an der Havel, Germany) according to the recommendations of the International Society for Clinical Electrophysiology of Vision (ISCEV)^2^. Digital fundus photographs were taken by a trained ophthalmic photographer using a confocal white-light fundus imaging system (EIDON, CenterVue, Padua, Italy). FAF and OCT scans were performed using the Spectralis OCT2 (Heidelberg Engineering, Heidelberg, Germany). The FAF images were acquired using an excitation wavelength of 488 nm and emitted fluorescence signals were detected between 500 nm and 700 nm using the confocal scanning laser ophthalmoscope component of this device. A 30° × 30° field was imaged using the high-resolution mode, with a resolution of 1536 × 1536 pixels. For OCT, volume scans were performed using 49 B-scans of the central 20° × 20° area, with 25 frames averaged for each B-scan.

*Audiological and Phoniatric evaluation*

Audiological tests included: Otoacoustic Emission (OAE), in terms of Transient-Evoked Otoacoustic Emission (TEOAE), for the evaluation of inner ear damage; Auditory Brainstem Responses (ABR), recorded in the frequency range between 2-4KHz, with stimulus parameters characterized by click of 0/1 ms, alternative polarity and rate of 21/s; liminar pure tone audiometry in the frequency range between 250 Hz-8000 Hz with evaluation of the Pure Tone Average (PTA); Impedance test with tympanometry + stapedial reflexes, i.e. the contraction of the stapedial muscle in response to an auditory stimulus (at ipsi and contra) at 500 Hz-1000 Hz-2000 Hz and 4000 Hz.

ABR and TEOAE are objective tests normally used for universal newborn hearing screening programs and therefore they are performed very early, during the first days/months of life. For the initial screening, as by protocol, infants are tested with TEOAE 24 hrs after birth and another screening is conducted for infants who fail the first-stage screening. They are retested within 10-15 days after birth by TEOAE and, in case of fail responses, the newborns are evaluated at the age of 3 months by ABR. An early diagnosis and immediate intervention play an important role in the development and prognosis of children with hearing loss and decrease the impact of the condition on the child's social, emotional, intellectual and linguistic development.

Auditory Brainstem Responses were evaluated with standard parameters. Three chloride silver electrodes were placed in the vertex (active), mastoid (right or left) and forehead (ground) positions. Electrode impedances were maintained at ≤ 6 kΩ. Stimuli for auditory brainstem response recording were digitized at a rate of 20 kHz,and presented over headphones. A conventional method of alternating click stimulus polarity was used to reduce stimulus artefacts in the average waveforms. Band-pass filtered the signals between 30 and 3000 Hz. Stimuli (clicks) were presented monaurally starting from 90 dB NHL, then gradually lowering the intensity. The average waveform was focused on a period extending from 10 ms before the stimulus to 10 ms after the stimulus. The presence/absence of the V wave, its morphology and its latency, at a pre-determined intensity of stimulation, are considered the most important parameters which help physicians to identify the presence and type of any hearing loss.

Pure-tone audiometry is the most common procedure used to evaluate hearing sensitivity, and it can determine the degree and type of hearing loss. The test was performed by using a standard two channel clinical audiometer (Resonance r27a) through the use of calibrated headphones in a silent booth. Air conduction pure tone thresholds at 250 Hz - 500 Hz - 1 kHz - 2 kHz - 4 kHz - 8 kHz and bone conduction thresholds (250 Hz to 4 kHz) were tested. Pure Tone Average (PTA) was calculated specifically at 500 Hz, 1000 Hz, 2000 Hz and 4000Hz. The audiometer/headphones were calibrated to ISO standards (International Organization for Standardization^3^.

The impedance analysis to evaluate the middle ear functioning was performed according to the guidelines of the American Speech-Language-Hearing Association. For this test, tympanometry and the acoustic reflexes were performed by the use of specific Middle Ear Analyzer (Resonance r26m diagnostic).

Phoniatric assessment was performed by a trained phoniatrician, specialized in evaluation, diagnosis and rehabilitation of speech, language and learning disabilities in childhood. This clinical examination consists in performing specific and validated Italian Standardized Tests, divided by age, able to underline the presence of alterations of various degrees in terms of communication and speech disorders (BVL 4-12)^4^, memory deficits (visual memory, working memory etc) ^5^ and alterations of the non-verbal intelligence quotient ^6^ which can negatively impact speech capacity and learning skills.

*Neuropsychological evaluation*

The following tests and questionnaires were administered:

1. the short version of Wechsler Intelligence Scale for Children (4th Edition) adapted for Italy (WISC-IV-Italian) which is an individually administered and norm-referenced instrument designed for measuring intelligence^7,8^. The WISC-IV-Italian contains four index scores. The Verbal Comprehension Index (VCI) includes the Vocabulary, Similarities, and Comprehension subtests; the Perceptual Reasoning Index (PRI) includes the Block, Design, Picture Concepts, and Matrix Reasoning subtests; the Working Memory Index (WMI) includes the Digit Span and Letter–Number Sequencing subtests; and the Processing Speed Index (PSI) includes the Coding and Symbol Search subtests. A total score relative to IQ was also obtained.
2. the CDI questionnaire (Children’s Depression Inventory), completed by the child, ^9^ that assesses symptoms of depression within the past two weeks and yields a total depression score.
3. the SCARED questionnaire ^10^ which is a 41-item scale that aims to indicate how children felt in the last three months. “Not True or Hardly Ever True,” “Somewhat True or Sometimes True,” and “Very True or Often True” are the response options. Reports from the child (SCARED-C) and parent’s (SCARED-P) can be used. The cut-off point of SCARED is suggested as 25.

*Dermatological evaluation*

Dermoscopy of nail plates (onychoscopy) and hair (trichoscopy) were performed through both handled (DermLite DL 200 Hybrid, 3Gen, LLC, Dana Point, Calif., USA) and digital dermoscopy lens (DermLite Foto lens, 3Gen, LLC, Dana Point, Calif., USA) coupled with a digital camera (Nikon Coolpix 3100; Nikon Corp., Tokyo, Japan)]. As adjuvant technique supporting trichoscopy, microscopic examination of scalp hair was performed through an optical microscope (Leica microsystems).

*Clinical exome sequencing and segregation analysis*

Genomic DNA was extracted from peripheral blood using the DNeasy Blood & Tissue Kit (QIAGEN) according to the manufacturer’s instructions. DNA was quantified on Nanodrop and integrity was visually assessed on an agarose gel. Libraries for clinical-exome sequencing were prepared using the ClearSeq Inherited Disease Panel (Agilent) according to the manufacturer's instructions. Libraries were run on a NextSeq500 sequencing platform (Illumina inc., San Diego, CA, USA) in collaboration with the NGS Core at TIGEM. Sequencing data were analysed in collaboration with the Bioinformatics Core at TIGEM using an in-house developed pipeline ^11,12^. Variants were filtered for Minor Allele Frequence (MAF≤0,01) in population databases (e.g. gnomAD, the ExAC database, the 1000 Genomes project etc). For variant interpretation, the retained SNVs were manually curated to assess their clinical significance taking into consideration the predicted functional consequences of the SNVs, *in silico* predicted pathogenicity parametres as well as their frequency in an internal database of sequencing variants in order to discard variants commonly encountered in the local population. The alignments at candidate positions were visually inspected using the Integrative Genomics Viewer (IGV). *In silico* predictions of pathogenicity of the missense variants from the Polymorphism Phenotyping v2 (Polyphen-2; <http://genetics.bwh.harvard.edu/pph2/>) ^13^, Sorting Intolerant from Tolerant (SIFT; <http://sift.bii.a-star.edu.sg/>)^14^ and Combined Annotation-Dependent Depletion (CADD; http://cadd.gs.washington.edu/)^15^ tools were considered. Selected variations were validated by Sanger sequencing in the patient and parents to assess proper segregation.

1. Iannaccone A, Kritchevsky SB, Ciccarelli ML *et al*: Kinetics of visual field loss in Usher syndrome Type II. *Invest Ophthalmol Vis Sci* 2004; **45:** 784-792.

2. McCulloch DL, Marmor MF, Brigell MG *et al*: ISCEV Standard for full-field clinical electroretinography (2015 update). *Doc Ophthalmol* 2015; **130:** 1-12.

3. Standardization IOf: Acoustics: reference zero for the calibration of audiometric equipment Part 1, Reference equivalent threshold sound pressure levels for pure tone and supra-aural earphones, 2004.

4. Marini A, Marotta L, Bulgheroni S, Fabbro F: Batteria per la Valutazione del Linguaggio in Bambini dai 4 ai 12 anni. *Firenze, Italy: Giunti OS* 2015.

5. Mammarella IC, Toso C, Pazzaglia F, Cornoldi C: *BVS-Corsi. Batteria per la valutazione della memoria visiva e spaziale. Con CD-ROM*. Edizioni Erickson, 2008.

6. Hammill DD, Pearson NA, Wiederholt JL: *Test TINV. Test di intelligenza non verbale*, Vol. 19. Edizioni Erickson, 1998.

7. Wechsler D: Wechsler intelligence scale for children–Fourth Edition (WISC-IV). *San Antonio, TX: The Psychological Corporation* 2003.

8. Orsini A, Pezzuti L, Picone L: Wechsler Intelligence Scale for Children IV Edizione Italiana. *Florence, Italy: Organizzazioni Speciali* 2012.

9. Kovacs M: *Children's depression inventory*. Multi-Health Systems North Tonawanda, NY, 1992.

10. Birmaher B, Khetarpal S, Brent D *et al*: The Screen for Child Anxiety Related Emotional Disorders (SCARED): scale construction and psychometric characteristics. *J Am Acad Child Adolesc Psychiatry* 1997; **36:** 545-553.

11. Di Iorio V, Karali M, Brunetti-Pierri R *et al*: Clinical and genetic evaluation of a cohort of pediatric patients with severe inherited retinal dystrophies. *Genes* 2017; **8**.

12. Musacchia F, Ciolfi A, Mutarelli M *et al*: VarGenius executes cohort-level DNA-seq variant calling and annotation and allows to manage the resulting data through a PostgreSQL database. *BMC Bioinformatics* 2018; **19:** 477.

13. Adzhubei I, Jordan DM, Sunyaev SR: Predicting functional effect of human missense mutations using PolyPhen-2. *Current protocols in human genetics* 2013; **Chapter 7:** Unit7 20.

14. Kumar P, Henikoff S, Ng PC: Predicting the effects of coding non-synonymous variants on protein function using the SIFT algorithm. *Nature protocols* 2009; **4:** 1073-1081.

15. Rentzsch P, Witten D, Cooper GM, Shendure J, Kircher M: CADD: predicting the deleteriousness of variants throughout the human genome. *Nucleic Acids Res* 2019; **47:** D886-D894.
